# Exploring willingness to pay for health insurance and preferences for a benefits package from the perspective of women from low-income households of Karachi, Pakistan

**DOI:** 10.1186/s12913-021-06403-6

**Published:** 2021-04-23

**Authors:** Shifa Salman Habib, Shehla Zaidi

**Affiliations:** 1 Community Health Solutions, 9th Floor, Al-Tijarah Building, Main Shahrah-e-Faisal, Karachi, Pakistan; 2 Department of Community Health Sciences, The Aga Khan University, National Stadium Road, Karachi, Pakistan

**Keywords:** Universal health coverage, Health insurance, Low-middle income countries

## Abstract

**Background:**

Achieving universal health coverage (UHC) and reduction in out of pocket (OOP) expenditures on health, is a critical target of the Sustainable Development Goals (SDG). In low-middle income countries, micro-health insurance (MHI) schemes have emerged as a useful financing tool for laying grounds for Universal Health Coverage. The aim of this study was to provide evidence for designing a feasible health insurance scheme targeted at urban poor, by exploring preferences for an insurance benefits package and co-payments among women from low-income households in Karachi, Pakistan.

**Methods:**

This was a descriptive cross-sectional study, conducted using household surveys between July–August 2015. A total of 167 female beneficiaries of *Benazir Income Support Programme* (BISP), a large-scale cash transfer scheme targeted at low-income households, were recruited in Karachi through a mix of convenience and snowball sampling. Hypothetical insurance benefits packages for a prospective health insurance scheme were formulated to capture respondents’ preferences for health insurance benefits package and co-payments. All data was analyzed using Stata (version 13).

**Results:**

Respondents reporting expenditure on OPD and hospitalization in the last 2 weeks were 93.4 and 11.9% respectively. The highest median expenditure was incurred on medicines. Out of the proposed benefits package, a majority (53%) of the study participants opted for the comprehensive benefits package that provided coverage for emergency care, hospitalization, OPD consultation, diagnostic tests and transportation. For the co-payment plan, 38.9% participants preferred no co-payments that is 100% insurance coverage of medicines followed by hospitalization (25.9%). Nearly half of the respondents (49.4%) chose outpatient consultation for 50% co-payment. A majority of the participants (65.3%) agreed to 100% co-payment for the transportation cost.

**Conclusion:**

Health insurance schemes can be introduced in urban areas, against collection of micro-payments, to prevent low-income households from facing financial catastrophe. A comprehensive benefits package covering emergency care, hospitalization, OPD consultation, diagnostic tests and transportation, is the most preferred among low-income beneficiaries.

## Background

Achieving universal health coverage (UHC), including provision of financial protection and reduction in out of pocket (OOP) expenditures on health, is a critical target of the Sustainable Development Goals (SDG) [1]. In low-middle income countries (LMIC), micro-health insurance (MHI) schemes have emerged as a useful financing tool for laying grounds for UHC [2, 3]. MHI is voluntary health insurance system that pools funds from members of a community, or a socio-economic organization, to ensure access to health care without facing adverse financial consequences [4]. MHI schemes are often implemented at the local level, targeting low-income households, and particularly those employed in the informal sector. These schemes have been associated with reduction in catastrophic health expenditures, OOP expenditure, household borrowings and protection of household assets in the beneficiary households [3].

In Pakistan, OOP expenditure accounts for 58% of all healthcare costs [5]. Health insurance can be a useful alternative to OOP expenditure and resultant financial consequences. However, insurance penetration remains low [6]. Since 2005, at least five micro-insurance schemes, have been introduced in Pakistan, primarily aimed at covering the costs of hospitalization [2]. In 2015, Pakistan’s federal government also launched a national health insurance initiative, *Sehat Sahulat Programme* which currently covers hospitalization for 6.7 million households, across 86 districts in the country [2]. These insurance schemes have been marked by poor utilization, secondary to low insurance literacy among the beneficiaries and lack of empaneled private providers ([2], https://www.aku.edu/news/Pages/News_Details.aspx?nid=NEWS-002320).

Designing a benefits package which is affordable and acceptable for the target beneficiaries has proven to be challenging for many insurance schemes in LMICs [7]. The scope of benefit package offered by an insurance scheme is a critical determinant of the community response to its introduction, acceptability, enrollment and overall sustainability [8–11]. Disagreement of the target groups with the insurance benefits package- example coverage of emergencies, outpatient care, medicines, transport cost- can result in low insurance scheme uptake and utilization [9, 12].

In order to improve the utilization and impact of health insurance schemes in LMICs it is of critical importance to examine the feasibility of implementing such programmes. Questioning of potential beneficiaries using hypothetical scenarios, can provide relevant information on beneficiary preferences on benefit packages, premiums and willingness to pay for insurance schemes, that can serve to strengthen the programme design and implementation strategies. This approach has been applied widely in African countries and India and have elicited strong willingness to pay and preferences for a comprehensive benefits package [13–16].

The primary aim of our study was to generate evidence for feasibility of a health insurance scheme targeted at the urban poor, from the perspective of beneficiaries. Using hypothetical insurance scenarios, this paper examines the preferences of females from low-income households, for an insurance benefits package and premiums for fund pooling, as opposed to out-of-pocket spending. The paper also reviews estimated magnitude and distribution of household out of pocket expenditure on health care among the urban, low-income households.

Our study attempts to provide lessons for policy makers for designing an affordable health care financing system targeted at the urban low-income population for progression towards UHC. The evidence presented in this paper may also inform the design and scale-up of *Sehat Sahulat Programme*, National Health insurance scheme in Pakistan to maximize its acceptability, uptake and utilization for the target populations, whilst also ensuring longer-term financial sustainability.

## Methods

### Study setting

This study was carried out in Karachi, the largest and most populous metropolitan city in Pakistan and the capital of Sindh province, with an estimated population of 23 million [17]. According to World Bank estimates, 22% of the population lives below the poverty line of $1.25 [18]. Karachi was selected for the study as an urban center may be a good starting point for an MHI scheme, where there is higher ability to pay for premiums, higher proportion of formal workforce whose salaries can be contributed towards an insurance pool and greater availability of health infrastructure.

### Study design

A descriptive cross-sectional study design, using household surveys, was utilized for the study. This was to provide an accurate description of OOP expenditure, health seeking patterns and preference for health insurance benefits package within the limited time frame of the study.

### Output variables

The following output variables were measured using the household surveys

- Health care utilization across levels of care (across private and public providers)
- OOP expenditure along the lines of hospitalization, consultation, drugs, diagnostic tests and transportation
- Willingness to enroll in a health insurance scheme.
- Preference for the type of health benefits package and level of co-payments

### Hypothetical MHI scheme

A hypothetical insurance benefits package for a prospective health insurance scheme was formulated to capture respondent’s willingness to pay for health insurance (Table 1). The costs of the proposed packages had been arrived at by carrying out desk review of MHI schemes operational in Pakistan and in other Asian countries like India, Bangladesh and China [19]. Presenting scenarios simplifies understanding of hypothetical programs such as MHI schemes which was a new concept for most of the respondents. A detailed explanation of the benefit packages was given to the respondents. For the context of our study, the benefit package referred to the range of services an individual will be willing to pay for if he or she is enrolled in the health insurance programme. All costs were given to respondents in the local currency (PKR) and converted into US dollars (US$) for presenting in this paper, as per the exchange rate in 2015.

**Table 1:** The hypothetical benefits packaged included in the survey

Package	Price/person/annum (PKR/US$)	Benefits
Package 1	PKR 150/US$1.4	• Accident & emergency
Package 2	PKR 250/ US$ 2.4	• Accident & Emergency • Hospitalization
Package 3	PKR 500/ US$ 4.8	• Accident & Emergency • Hospitalization • OPD (consultation, tests) • transport

### Sampling

The female beneficiaries of *Benazir Income Support Programme* (BISP), a large-scale cash transfer scheme targeted at low-income households, were recruited in Karachi through a mix of convenience and snowball sampling [20]. A household was identified as vulnerable and eligible to participate in the study, if at least one female in the household was enrolled with BISP and reported the incidence of a household member falling ill in the last 2 weeks on verbal screening carried out by the interviewer. On the days of the survey, the sampling frame considered were all the women enrolled with BISP and present at the BISP customer service and complaint office. Due to time and budgetary constraints, the BISP office was selected as the study location to ensure convenience of locating BISP beneficiaries under one-roof. At its core, the BISP, operational since 2008, is an Unconditional Cash Transfer (UCT) providing quarterly cash payments directly to female beneficiaries within households identified by implementing a World Bank poverty scorecard survey [21]. The poverty scorecard is based on a proxy means testing (PMT), which involves using proxies of income such as personal or family characteristics (example ownership of car). All households identified below the PMT threshold of 16.17 were eligible to participate [21]. The last impact evaluation by OPM was conducted in 2019 and evaluated its impact on poverty reduction, child nutrition, education and women empowerment [22]. BISP is one of the largest social cash transfer programmes in the region, covering 5 million households, and now integrated within the *Ehsaas Programme*, a broader poverty alleviation programme by the Government of Pakistan [23].

The PSLM (Pakistan Living Standard Measurement) survey 2012–13 [24] was used to estimate the incidence of illness for sample size calculation. During the reference period of 2 weeks prior to the PSLM survey an average of 10.98% of the population had fallen sick or injured (10.92 in urban and 12.62 in rural parts of Karachi). A sample size of 167 was determined, using 95% confidence level and precision level of 5%, also accounting for 10% non-response rate.

Recruitment of BISP beneficiaries as study participants was to ensure that they have been identified below the poverty cut-off in the poverty score card implemented by BISP. The 2 weeks recall period for an episode of illness, was chosen in line with the reporting standards of PSLM survey 2012–13.

### Data collection

The cross-sectional data was obtained using a pre-structured questionnaire translated into local languages *Urdu* and *Sindhi*. The questionnaire was designed after careful review of data collection processes and outcome variables stated in other studies on the subject [7–16, 19, 23–28]. Data collection was completed during July–August 2015. The interviewers were trained over a period of 2 weeks to ensure their command on administering the questionnaire and understanding health insurance. In addition, all the interviewers were trained and tested to phrase the questions in a uniform and consistent manner. A pre-test was done on 16 participants (10% of the sample size) to validate cognitive suitability. After the pre-test, the option of transport was included in the response options and insurance package.

### Statistical analysis

All data was analyzed using Stata version 13.0. Descriptive statistics were computed for demographic data, healthcare utilization, household expenditures on healthcare, the participants’ willingness to pay and their choice of co-payment with respect to service to be used. We also conducted comparison of categorical socio-demographic variables with the choice of benefits package.

### Ethical approval

The study was approved by the Ethical review Committee at the Aga Khan University. Written and verbal informed consent, in the local language, was acquired from all respondents. De-identified data was utilized for the final data analysis.

## Results

A total of 167 women participated in the study. Table 2 summarizes the general characteristics of the households and female beneficiaries included in the survey. The median size of the participating household size was 8. About a third of the households had a monthly income of less than PKR 10,000 (US$ 96). Majority of the respondents had received no education. About 20% of the female beneficiaries were working as informal workers whereas the majority served as housewives.

**Table 2:** Socio-demographic profile of the respondents

Household characteristics	Frequency/n	% (proportion)
**Median household size**	8	
**At least one child under 5**	114	68.3
**At least elderly over 65**	35	21.0
**Monthly income**		
< PKR 10000/ US$ 96.2	53	31.7
PKR 10000–15,000/ US$ 96–144	87	52.1
PKR 15000–20,000/ US$ 144–192	27	16.2
**Residence type**		
Self-owned	113	67.7
Rented	49	29.3
Others	5	3.0
**Female characteristics**		
**Age**		
20–29 years	13	7.8
30–39 years	66	39.5
40 years and above	88	52.7
**Education**		
No education	128	76.6
Primary	22	13.2
Secondary	10	6.0
Matriculation	7	4.2
**Occupation**		
Domestic workers	33	19.8
Housewives	134	80.2

### Healthcare Expenditure & Utilization patterns

**Fig. 1:**
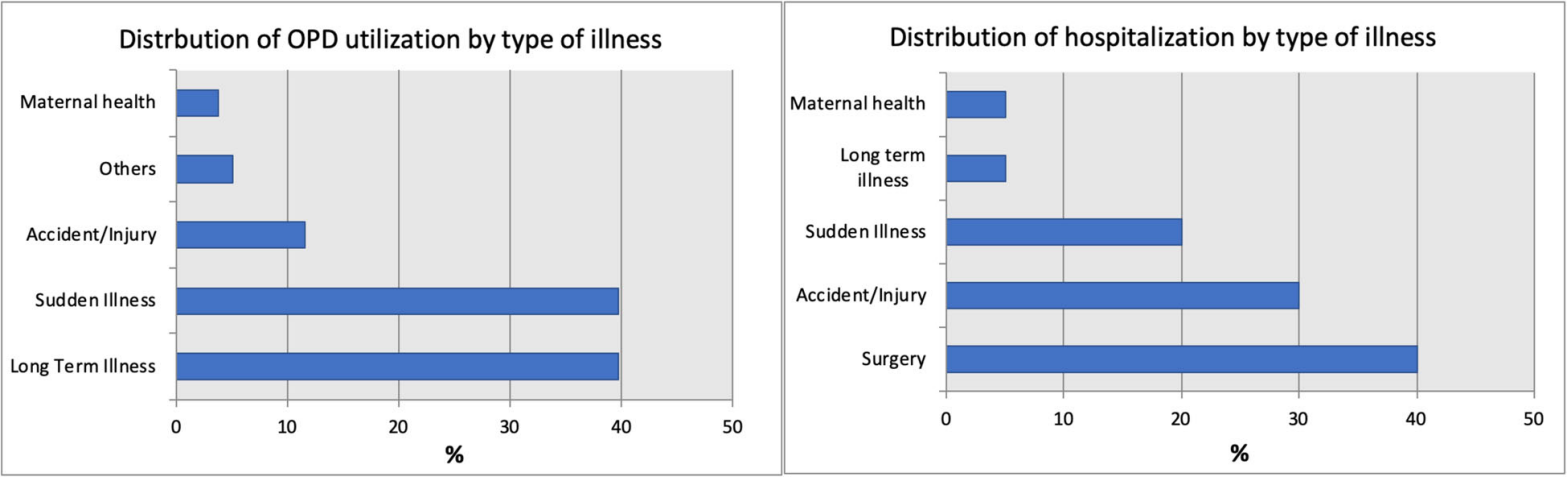
Distribution of OPD and hospitalization utilization by type of illness

A majority (93.4%) of participating households incurred OPD expenditures or reported at least one OPD visit in the last **2** weeks (Table 3). Most of the people availed OPD services for chronic and acute illnesses, (40%) (Fig. 1). Only 12% households reported an event of hospitalization for a household member, with surgical procedures accounting for the majority of hospitalization cases, followed by accident and emergency (Fig. 1).

**Table 3:** Disaggregated healthcare expenditures by expenditure categories, occurring over last 2 weeks in the sampled households

	Number of cases incurring expenditure (n)	Proportion of cases incurring expenditure (%)	Median expenditure in PKR/ US$	Proportion of sum of all OPD/hospital expenditures
**Out patient**				
Total	156	93.4	PKR 1410/ US$ 14	100
Consultation	110	65.8	PKR 100/ US$ 1	7.37
Medicines	152	91	PKR 850/ US$ 8	66.57
Diagnostic tests	59	35.3	PKR 500/ US$ 5	14.31
Transport	117	70	PKR 300/ US$ 3	14.4
**Hospitalization**				
Total	20	11.9	PKR 4500/ US$ 43	100
Hospital expenditure	20	11.9	PKR 3750/ US$ 36	89.72
Transport	19	11.3	PKR 600/ US$ 6	10.82

Disaggregation of healthcare expenditure across cost categories, shows that the cost of medicines is contributing towards the highest OOP expenditure and is also most commonly faced by 91% households (Table 3). Diagnostic tests, transport and doctor’s consultation accounted for a median expenditure of PKR 500, 300 and 100 respectively. Only 20 out 167 households reported hospitalization expense in the last 2 weeks. Median in-patient hospital expenditure was found to be PKR 3750/ US$ 36, compared to median expenditure of PKR 1410/ US$ 14 for outpatient visit.

### OOP by public versus private sector

71% of outpatient visits were at private health facilities, equally distributed over private clinics and hospitals. The classification of private health facilities included commercial for-profit as well as philanthropic facilities. The remining 29% of out-patient cases were seen at government hospitals, with no reporting of cases at government primary care clinics. Out-patient expenses at government health facilities were higher, with a median expenditure of PKR 2000/US$ 19.2, in comparison to PKR 1300/ US$ 12.5 at private hospitals and PKR 1250/ US$ 12 at private clinics. (Table 4). The OOP expense of out-patient visits at government hospitals was driven up mainly by medicines and diagnostics which were not provided at government hospitals and had to be purchased at private outlets. The comparison between public and private facilities does not take into account the comparability of cases*.*

**Table 4:** Disaggregated OPD expenditure at various health care facilities

	Government Hospital		Private Hospital		Private Clinic	
	Number of cases/n	Median expenditure/PKR/ US$	Number of cases/n	Median expenditure/PKR/ US$	Number of cases/n	Median expenditure/PKR/ US$
Total	45	PKR 2000/ US$ 19	59	PKR 1300/ US$ 12.5	52	PKR 1250/ US$ 12
Consultation	14	PKR 100/ US$ 1	47	PKR 100/ US$ 1	48	PKR 100/ US$ 1
Medicines	42	PKR 1000/ US$ 9.6	58	PKR 850/ US$ 8	52	PKR 600/ US$ 6
Diagnostic Tests	23	PKR 1000/ US$ 9.6	17	PKR 400/ US$ 4	19	PKR 500/ US$ 5
Transport	39	PKR 500/ US$ 4.8	46	PKR 400/ US$ 4	32	PKR 200/ US$ 2

### Preference for benefits package

**Fig. 2:**
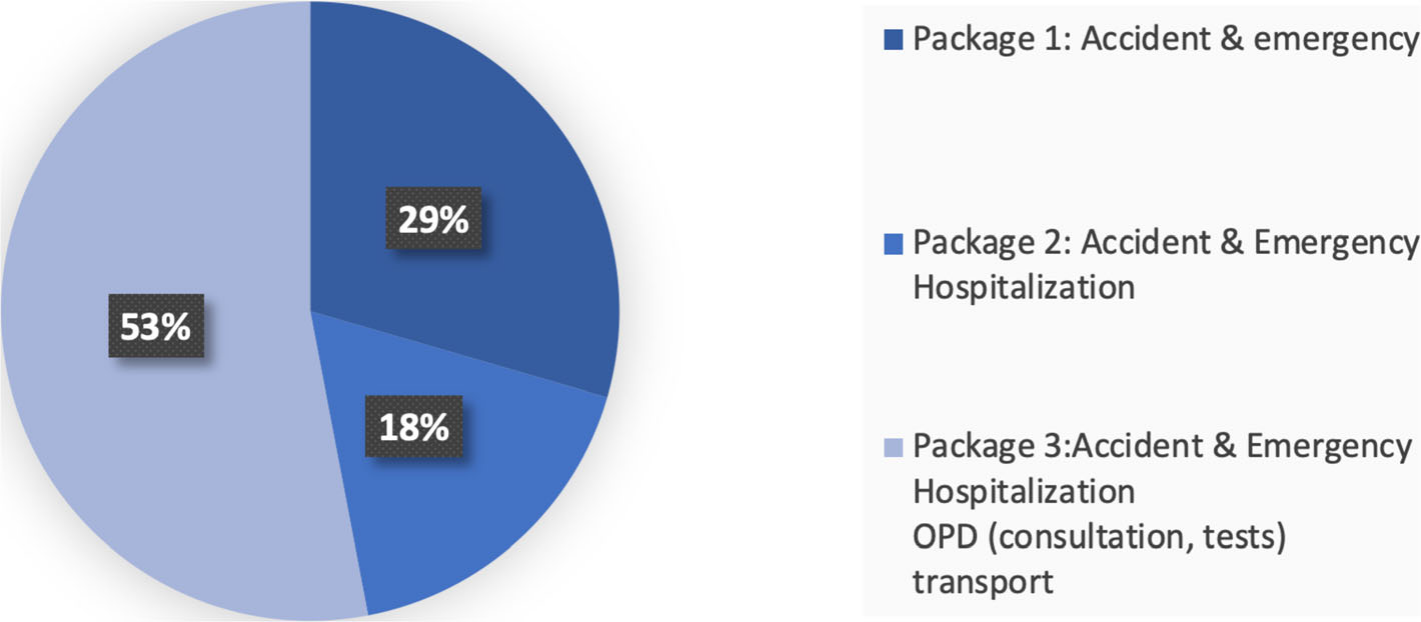
Preference for insurance benefits package

Figure 2 summarizes participants’ characteristics according to their choice of hypothetical insurance package. More than half of the participants (53%) opted for the third package with the annual payment of PKR 500 (US$ 5) per person per household, that included emergency care, inpatient care due to any cause, outpatient care, and transportation cost. A majority of the households with a child under 5, monthly income of PKR 10000–15,000/ US$ 96–144 (second highest income bracket) and living in self-owned accommodation selected package 03 (Table 5). The second highest preference (29%) was seen for package 1 offering accident and emergency.

**Table 5:** Socio-demographic profile of the respondents by type of package selection

Household characteristics	Package 1	Package 2	Package 3
	N (%)	N (%)	N (%)
Total	49 (29.5)	29 (17.5)	88 (53.0)
Median household size	9	8	8
At least one child under 5	36 (31.6)	20 (17.5)	58 (50.9)
At least one elderly over 65	11 (31.4)	4 (11.4)	20 (57.2)
**Monthly income**			
< PKR 10000/ US$ 96.2	16 (30.2)	10 (18.9)	27 (50.9)
PKR 10000–15,000/ US$ 96–144	28 (36.6)	16 (18.6)	42 (48.8)
PKR 15000–20,000/ US$ 144–192	5 (18.5)	3 (11.1)	19 (70.4)
**Residence type**			
Self-owned	28 (25.0)	18 (16.1)	66 (58.9)
Rented	20 (40.8)	9 (18.4)	20 (40.8)
Others	1 (20.0)	2 (40.0)	2 (40.0)
**Female beneficiary characteristics**			
**Age**			
20–29 years	6 (31.6)	4 (21.0)	9 (47.4)
30–39 years	21 (31.4)	11 (16.4)	35 (52.2)
40 years and above	22 (27.5)	14 (17.5)	44 (50.0)
**Education**			
No education	40 (31.3)	21 (16.4)	67 (52.3)
Primary and above	9 (23.7)	8 (21.0)	21 (55.3)
**Employment status**			
Employed for remuneration	8 (24.2)	6 (18.2)	19 (57.6)

**Fig. 3:**
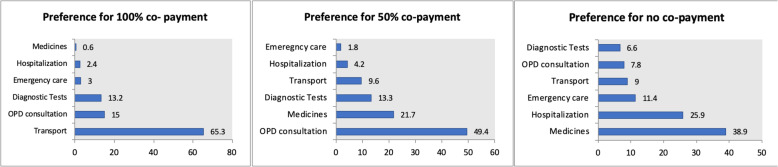
Preference for various degrees of insurance coverage and co-payments

With regards to participants’ preference for co-payment for the various health services, a majority (38.9%) participants preferred no co-payments that is 100% insurance coverage of medicines followed by hospitalization (25.9%) (Fig. 3). Nearly half of the respondents (49.4%) chose outpatient consultation for 50% co-payment. A majority of the participants (65.3%) agreed for 100% co-payment for the transportation cost (Fig. 3).

## Discussion

This is the first study from Pakistan, that contributes towards feasibility of designing a health insurance scheme targeted at low-income urban households by examining their willingness to enroll in such a scheme, preferences for the insurance benefits package and co-payments. We also examined healthcare expenditures and utilization patterns of the households which may influence their decision-making regarding insurance enrollment. This study was also unique in capturing preferences of potential women beneficiaries from low-income households, a frequently neglected group in the design of health insurance schemes. Our study findings indicate that low-income urban households are willing to enroll in a health insurance plan that provides them with comprehensive coverage against health shocks, against small monthly payments.

We found that over a 90 % of the households had incurred OOP expenditure at the outpatient level in the last 2 weeks, with 50% of all OPD expenditure taking place at private facilities. Factors such as overcrowding, poor perception of quality, low responsiveness and difficult geographical access to government facilities may have shifted the consumer favor towards private facilities [29]. Consequently, about half of the respondents preferred at least 50% of OPD expenditures to be covered by the prospective health insurance scheme. This may be also explained by having women as study participants, who may have a higher utilization of OPD for services such as MNCH care.

Currently the MHI and other government supported health insurance initiatives in Pakistan are focused on hospitalization and have low uptake by the target population. Pakistan’s *Sehat Sahulat Programme* (SSP), the national health insurance initiative, also, like BISP cash transfer scheme, uses a poverty-based scorecard to offer insurance cover to the poorest citizens [2]. The scheme is currently marked by significant underutilization, with the current rate of utilization at 3%, potentially owing to low insurance literacy among the insured population, lack of coverage for outpatient and primary care services and low numbers of empaneled private providers. Other studies from Iran, India, and Kenya have identified lack of OPD coverage as a critical weakness of insurance schemes in these countries [30–32]. Our findings highlight the need for an urban primary health care (PHC) network as part of the health insurance scheme to cover outpatient services. There is also critical need to purchase health services from private hospitals, clinics and diagnostic centres to supplement government services for meeting the mandate of UHC. Strategic purchasing is one of the key policy instruments for UHC goals of equitable access and financial risk protection, as has been identified as one of the main drivers of Thailand’s universal health coverage scheme [33].

Majority (53%) of the respondents in our study chose hypothetical benefits package 03, which was the most costly but comprehensive package that includes emergency care, hospitalization, OPD consultation, diagnostic tests and transportation. This finding is consistent with previous studies where strong preferences were elicited for inclusion of high-cost health services such as surgical operations as well as low cost items such as consultation fees [13]. The extensive use of OPD by the respondents may have driven majority of them to choose a package that provides coverage for OPD expenses. A health insurance benefit packages covering all major causes of ill-health in a target community would ensure that the beneficiaries derive optimal benefit from insurance package and perceive it as good value for money.

We found that 38.9% of the study participants chose medicines as the entity of choice to be covered fully in the insurance benefits package so that no co-payment is required to be made at the time of obtaining medicines. This preference co-relates with our finding of high OOP expenditure in the line of medicines (median PKR 800/ US$ 8), which could be a financial burden on the low-income households. This is consistent with the NHA 2015–16, where the medicines accounted for majority (47.3%) of all OOP health expenditure in Pakistan and 45.1% in Sindh [5]. In low-middle income countries, irrational use of medicines, including self-medication and prescription medicines, frequently result in high expenses incurred on medicines [34].

Most (65.7%) people agreed to completely bear the transport expenses themselves without it being covered by insurance. Access to a healthcare facility and the associated transportation is potentially not inconvenient for the participating families, due to this being an urban sample.

It is important to note that while this study captures women perspectives towards health insurance, data from Demographic and Health Surveys (DHS) show that women are not involved in decisions concerning their own health in 50% or more of the households in LMICs [35]. Therefore, for enhancing insurance utilization, both men and women need to be educated on health insurance, benefits package, claims processing and mobilized for improved decision-making.

## Conclusions and policy implications

Our study explored the preferences of the low-income households for health insurance benefits package and co-payments. The study findings can potentially feed into the design and scale-up of MHI schemes in Pakistan and also *Sehat Sahulat Program* (SSP), the National Health Insurance scheme that envisions to improve access to healthcare for poorest segments of the country.

Our study highlights the potential of collecting micro-payments from participating households, particularly in the urban areas, to ensure greater financial sustainability of the health insurance schemes. Secondly, it also reviews beneficiary preferences for the benefits package which is most suitable to their requirements, for instance, OPD coverage. This could potentially improve uptake and utilization of the envisioned health insurance scheme.

Our study concludes that health insurance schemes can be introduced in urban areas, to prevent low-income households from facing impoverishment and financial catastrophe due to the burden of OOP payments.

### Limitations

This study was limited to cross sectional study design whereas a mixed methods study design could help in gaining an in-depth understanding of the choices for co-payments and the benefits package. Sample comprises respondents who had reported an episode of illness for a household member and therefore may be more inclined towards choosing a pre-paid insurance scheme due to OOP incurred. The information on preference for health insurance package can be compared with relevant premium levels for benchmarking only (as premiums may not reflect actual willingness to pay). In addition, the actual insurance enrollment could be lower as this study captures the perspectives of women who may not necessarily be the sole decision makers in their households.

## Data Availability

The datasets used and analysed for the study under review are available from the corresponding author on reasonable request.

## Abbreviations

UHC: Universal Health Coverage
OOP: Out of pocket
SDG: Sustainable Development Goals
MHI: Micro Health Insurance
LMIC: Low-Middle Income Countries
NHI: National Health Insurance
CHE: Catastrophic Health Expenditure
WTP: Willingness to pay
SSP: Sehat Sahulat Programme
BISP: Benazir Income Support program
UCT: Unconditional cash transfer
OPD: Outpatient department
CBHI: Community based Health Insurance
NIC: National Identity Card
ERC: Ethical review committee
PSLM: Pakistan Living Standard Measurement survey
NHA: National Health Accounts
